# It Is Always Early with Point-of-Care Ultrasound

**DOI:** 10.1155/2020/9431496

**Published:** 2020-04-04

**Authors:** Zouheir Ibrahim Bitar, Tamer Mohamed Zaalouk, Ossama Sajeh Maadarani, Ragab Desouky Elshabasy

**Affiliations:** Critical Care Unit, Ahmadi Hospital, Kuwait Oil Company, P.O. Box 46468, 64015 Fahaheel, Kuwait

## Abstract

A 56-year-old male was admitted to the emergency department for acute pulmonary edema and septic shock, yet no clear source of infection was noted upon physical examination. Due to his unstable condition, bedside ultrasound was performed. A heterogeneous mass in the liver was noted; hence, a tentative diagnosis of liver abscess was made. The abscess was confirmed by abdominal magnetic resonance imaging. Drainage of the abscess was attempted and guided by early ultrasound. This case highlights that point-of-care ultrasound, when performed by an ultrasound-capable critical care physician, can significantly decrease the time to diagnosis for septic patients.

## 1. Case File

A 56-year-old male presented to our hospital's emergency department complaining of acute shortness of breath and 1 week of malaise. He complained of nausea and vomiting in that period, with mild abdominal pain, but no diarrhea, cough, chest pain, or dysuria. He was known to have diabetes mellitus requiring insulin, hypertension, an old myocardial infarction followed by left anterior descending artery and right coronary artery stenting with borderline impaired systolic function 6 months prior, and dyslipidemia.

Upon examination, he had a Glasgow Coma Scale of 15, was shivering, hypotensive (mean arterial blood pressure 40 mmHg), tachycardic (140 beats per minute), tachypneic (34 breaths/min), hypoxic (peripheral oxygen saturation 82% on room air), and febrile (temperature of 38.9°C). An arterial blood gas on 6 L/min of oxygen through a BiPAP machine revealed a compensated acute metabolic acidosis, with a pH of 7.404, pCO2 15 mmHg, pO2 101 mmHg, HCO3 15.4 mmol/L, and lactate 4 mmol/L. The patient was in a hyperosmolar state with high blood sugar 30 mml/L and normal blood ketones. He was admitted to the critical care unit upon diagnosis of acute pulmonary edema and suspicion of sepsis. The Rapid Assessment of Dyspnea with Ultrasound protocol [1] was immediately started and showed depressed systolic function of the left ventricle, with cardiac index 2 mL/min/m^2^, and ultrasound of the chest showed severe bilateral ultrasonic B lines suggestive of acute pulmonary edema. Clinically, there was still no obvious source of infection: he had a bilateral pulmonary crepitation and there was no abdominal or costovertebral angle tenderness.

**Figure 1:**
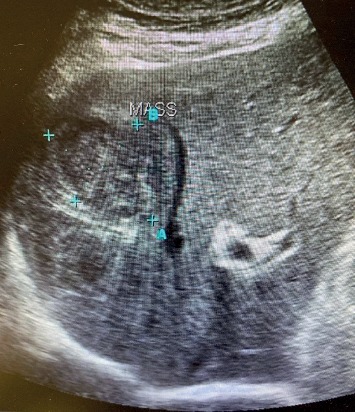
Ultrasound liver showing the heterogeneous mass suggestive of abscess.

**Figure 2:**
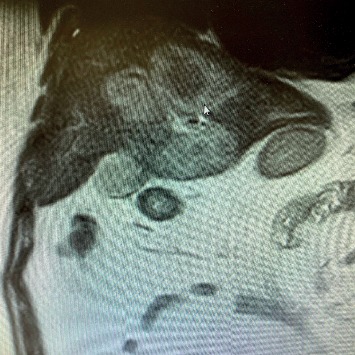
MRI abdomen showing T1 hypointense and T2 heterogeneous hyperintense thin-walled peripheral rim enhancing and intercommunicating cystic mass measuring predominately involving segments IV a, IV b, and I.

The patient's blood pressure was still low after dobutamine infusion followed by noradrenaline infusion and controlled intravenous fluid with insulin infusion; because the patient continued to be unstable, a bedside ultrasound examination for critically ill patients was performed to further look for a source of infection. Ultrasound revealed a heterogeneous mass seen in segments IV (at the level of the left portal vein of the left lobe), VIII and V (at the level of the right portal vein of the right liver lobe), and I (caudate lobe) of the liver with no obvious intrahepatic biliary dilatation. The largest mass measured 10 × 7 cm (Figure 1). A thickened wall of the gall bladder with gall stones and biliary mud was also seen suggestive of acute cholecystitis. A liver abscess was suspected. An abdominal magnetic resonance imaging scan was then performed, which confirmed the abscess with no obvious intrahepatic biliary dilatation; the mass measured 9.5 × 9.3 × 9.5 cm of volume 435 cc seen predominately involving segments IV and I (Figure 2). Piperacillin-tazobactam and metronidazole had already been administered. Urgent percutaneous ultrasound-guided drainage of the hepatic abscess revealed a thick purulent material, and then, with the help of the interventional radiologist, cholecystostomy was performed the next day; cultures later isolated *Enterococcus avium*. Cholecystostomy was performed because the patient was considered a high surgical risk for cholecystectomy. Abscess drainage and appropriate antibiotic therapy led to steady improvement, both clinically and on repeated imaging exams. The patient was discharged 2 weeks later with a stable condition, and laparoscopic cholecystectomy was performed 6 weeks later.

## 2. Discussion

Early recognition of septic shock and the initiation of appropriate antibiotics is the cornerstone treatment [2]. The optimal timing of source control is unknown but guidelines suggest no more than 6 to 12 hours after diagnosis since survival is negatively impacted by inadequate source control [3]. POCUS helps not only in the early identification of the source of infection but also in early interventions such as drainage or removal of the infectious source. However, because of the hemodynamic instability of septic shock patients [1], it is reasonable to carry out diagnostic and interventional ultrasound-guided procedures bedside.

Liver abscesses are the most common type of visceral abscess, and its risk factors include diabetes mellitus, underlying hepatobiliary or pancreatic disease and regular use of proton-pump inhibitors [4]. Ultrasound, computed tomography (CT), and magnetic resonance imaging are the diagnostic modalities typically used for identifying liver abscesses [5]. There is usually a delay of 1 week between the onset of symptoms and diagnosis, as most abscesses are nonspecific, and there are multiple occult sources of infection, such as silent gall bladder diseases and diverticulitis [6].

Streptococci, including *Enterococcus avium*, were the most common pathogen of pyogenic liver abscess in the United States [7]. Enteric gram-negative bacilli, particularly *K. pneumoniae*, are commonly identified pathogens in Asia and suggested an association with underlying colorectal cancer with a prevalence of colorectal cancer among pyogenic liver abscess patients 2.3 to 3.2 percent in long-term follow-up [8].

Percutaneous cholecystostomy in acute cholecystitis is indicated as the initial treatment, combined with antibiotics, for patients who are at high surgical risk such as being septic or critically ill [9]. Early cholecystostomy (<24 hours from symptom onset) was associated with a lower complication rate and a shorter hospital stay [10].

Identifying the source of infection can be challenging, yet important for managing the patient. A stepwise, structured POCUS approach helps to do so in a minimal amount of time.

Point-of-care ultrasound, in the hands of a critical care physician, significantly decreases the time to diagnosis for septic patients, potentially decreasing the time to source control.
